# New records and a new species of chewing lice (Phthiraptera, Amblycera, Ischnocera) found on Columbidae (Columbiformes) in Pakistan

**DOI:** 10.3897/zookeys.174.2717

**Published:** 2012-03-09

**Authors:** Saima Naz, Oldrich Sychra, Syed Anser Rizvi

**Affiliations:** 1 Department of Zoology, University of Karachi, Karachi, 75270, Pakistan; 2 Department of Biology and Wildlife Diseases, Faculty of Veterinary Hygiene and Ecology, University of Veterinary and Pharmaceutical Sciences, Palackého 1–3, 612 42 Brno, Czech Republic

**Keywords:** chewing lice, Columbidae, Pakistan, new records, new species, taxonomy

## Abstract

The chewing lice (Phthiraptera) of Columbidae (Columbiformes) from Pakistan are studied. Six species of chewing lice with new host records are recorded and one new species of the genus *Colpocephalum* is described from *Columba livia* in the Karachi region. All the columbid chewing lice from Pakistan are keyed out and the new species is illustrated and compared with the closest allied species.

## Introduction

The study of chewing lice in Pakistan has been neglected for many years, especially in the Sindh region of Pakistan. During 1940s to 1950s, Ansari published his work on lice from Pakistan, but his studies were restricted to Lyallpur (now Faisalabad), in the Punjab Province of Pakistan (Ansari 1947, 1951, 1955a–e, 1956a, b). Most of his work referred to the Punjab region of India (Ansari 1957a, b, 1958, 1959).

Lakshminarayana (1979) published a list of Mallophaga from India and its adjacent countries, listing only those chewing lice species that were reported by Ansari from Lyallpur, Pakistan.

After Ansari (1955b, 1958), no taxonomic studies have been carried out in Karachi, Pakistan. Here we present a key to species of columbid chewing lice of this region and describe a new species of the genus *Colpocephalum*. This new species is compared with the closest allied species of the genus.

## Material and methods

The chewing lice used in this study were preserved on microscopic slides using a standard method (Palma 1978) and mounted in Canada-balsam. Line diagrams were made using micro-ocular graticule with a light microscope. Collected species have been deposited in the Natural History Museum, University of Karachi (NHMUK), Pakistan and the Moravian Museum (MZM), Burno, Czech Republic.

### Abbreviations:

AL Abdominal Length

DHS Dorsal Head Seta

GL Genital Length

HL Head Length

ML Metathorax Length

MW Metathorax Width

PL Pronotal Length

PML Paramere Length

POW Preocular Width

PW Pronotal Width

TL Total Length

TW Temporal Width

## Results

### Chewing lice Species of Columbidae in Pakistan

*Bonomiella columbae* Emerson, 1957 – **New record**

*Campanulotes bidentatus* Scopoli, 1763 (Lakshminarayana 1979)

*Campanulotes compar* Burmeister, 1838 – **New record**

*Coloceras piageti* Johnston & Harrison, 1912 (Ansari 1947, Lakshminarayana 1979)

*Colpocephalum afrozeae* sp. n.

*Colpocephalum turbinatum* Denny, 1842 (Ansari 1951)

*Columbicola columbae* L. 1758 (Ansari 1947)

*Columbicola theresae* Ansari, 1955 (Lakshminarayana 1979)

*Columbicola tschulyschman* Eichler, 1942 – **New record**

*Hohorstiella lata* Piaget, 1880 – **New record**

*Hohorstiella modesta* Ansari, 1951 (Lakshminarayana 1979)

*Hohorstiella streptopeliae* Eichler, 1953 – **New record**

*Turturicola salimalii* Clay & Meinertzhagen,1937 – **New record**

### Key to the chewing lice species of Columbidae in Pakistan

**Table:** 

1	Maxillary palpi present; meso and metathorax separated	Amblycera, 2
–	Maxillary palpi absent; meso and metathorax fused, forming pteronotum	Ischnocera, 7
2	Postpalpal process present	*Hohorstiella*, 3
–	Postpalpal process absent	5
3	Head much broader than long; anterior head margin broadly convex; abdomen short and oval; three abdominal sternites (st. III–V) with thick setal brushes	*Hohorstiella modesta* (Ansari)
–	Head broader than long; anterior head margin relatively more convex; abdomen large and oblong; two abdominal sternites with setal brushes	4
4	Postpalpal process short; antennal segment II small and rounded; prosternal plate small; abdominal sternite IV–V with thin setal brushes; vulval margin wide with thin short to long setae	*Hohorstiella lata* (Piaget)
–	Postpalpal process long; antennal segment II large and globulate; abdominal sternite III–IV with dense setal brushes; vulval margin narrow with thin microsetae to short fine setae	*Hohorstiella streptopeliae* Eichler
5	Head without ocular and occipital carinae; femur III and abdominal sternites without ctenidia	*Bonomiella columbae* Emerson
–	Head with ocular and occipital carinae; femur III and abdominal sternites with fine ctenidia	*Colpocephalum*, 6
6	Femur III and abdominal sternite III with two fine ctenidia on each; male genital sclerite large, with short and fine latero–posterior points; penis short; female subgenital plate with medially short, stout setae	*Colpocephalum afrozeae* sp. n.
–	Femur III and sternite III with three ctenidia on each; male genital sclerite with long and slightly curved latero–posterior points; penis long; female subgenital plate with lateral tufts of setae	*Colpocephalum turbinatum* Denny
7	Head circumfasciate; temples large or broad, angulated	8
–	Head non–circumfasciate; temples short and rounded	10
8	Antennae dimorphic; scape very enlarged in male	*Coloceras piageti* (Johnston and Harrison)
–	Antennae monomorphic	*Campanulotes*, 9
9	Female larger in size, not less than 1.58 mm long; ventral median setae on sternites VI and VII absent	*Campanulotes bidentatus* (Scopoli)
–	Female smaller in size, not more than 1.34 mm long; ventral median setae on sternites VI and VII present	*Campanulotes compar* (Burmeister)
10	Median head setae blade-like, on anterior dorsal plate; anterior dorsal plate divided medially; preantennal width narrow	*Columbicola*, 11
–	Median head setae not blade-like, on anterior dorsal plate; anterior dorsal plate complete; preantennal width broad	*Turturicola salimalii* Clay and Meinertzhagen
11	Head length more than 0.55mm; posterior median head setae spike-like, shorter than anterior median head setae; male genitalia with triangular mesosomal plate, with groves directed towards median; female subgenital plate without lateral row of setae, grove with clear lateral indentations	*Columbicola theresae* Ansari
–	Head length less than 0.55mm; posterior median head setae hair like or spike like, equal or longer than anterior median head setae; male genitalia with medially divided mesosomal plate, with anterior grove, bearing pores in or out of the pigmented border; female subgenital plate with lateral row of setae, grove without indentations	12
12	Posterior median head setae hair-like and longer than anterior median head setae; male genitalia with relatively long, straight and posteriorly narrower parameres, mesosomal plate with shallow and narrow anterior grove, two pairs of pores present at mediolateral margins of mesosomal plate; female subgenital plate narrow with smooth posterior grove	*Columbicola tschulyschman* Eichler
–	Posterior median head setae spike-like, more or less equally long to anterior median head setae; male genitalia with short, stumpy parameres, curved inside outwards, mesosomal plate with large or deep anterior grove, anterior pair of mesosomal pores present at lateral margins within the dark pigmented borders; female subgenital plate relatively wider with wavy posterior grove, long and wide, bearing 4–8 pairs of medium to long setae	*Columbicola columbae* (L.)

### Suborder Amblycera Kellogg, 1896

**Family Menoponidae Mjöberg, 1910**

#### Bonomiella columbae

Emerson

http://species-id.net/wiki/Bonomiella_columbae

Bonomiella columbae Emerson 1957: 63, 1972: 37, Selim et al. 1968: 79, Hill and Tuff 1978: 308, 316, Price et al. 2003: 93, 303, 308.

##### Material examined.

2 females, on *Columba livia* (Gmelin); Pakistan: Karachi; 21-V-2004; leg. Naz.

New record from Pakistan.

#### Colpocephalum afrozeae sp. n.

urn:lsid:zoobank.org:act:CC7DD2BC-D82F-4E06-89E7-8C3EB8A5739F

http://species-id.net/wiki/Colpocephalum_afrozeae

Figs 1 12

##### Holotype.

male, on *Columba livia* (Gmelin); Pakistan: Karachi; 20-VII-2006; leg. Naz, S.

##### Paratype.

8 males, 12 females, on *Columba livia* (Gmelin); Pakistan: Karachi; 20-VII-2006; leg. Naz, S.

##### Other material.

6 nymphs, on *Columba livia* (Gmelin), with data as above.

##### Type host.

*Columba livia* (Gmelin) (Columbiformes: Columbidae).

##### Measurements.

**Figures 1–10.**
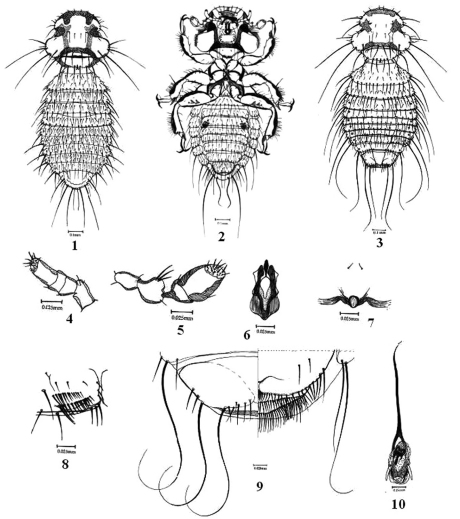
*Colpocephalum afrozeae* sp. n.**1** male dorsal view **2** male ventral view **3** female dorsal view **4** maxillary palp **5** antenna; **6** hypopharynx **7** prosternal plate **8** sternite IV with ctenidia **9** female terminalia ventral view **10** male genitalia

TL: male 1.242 (1.24–1.245) (Figs 1–2), female 1.330 (1.285–1.375) (fig. 3); HL: male 0.287 (0.286–0.288), female 0.302 (0.30–0.305); POW: male 0.318 (0.315–0.332), female 0.347 (0.345–0.35); TW: male 0.45 (0.445–0.455), female 0.492 (0.48–0.505); PL: male 0.12 (0.11–0.13), female 0.137 (0.135–0.14); PW: male 0.288 (0.255–0.322), female 0.332 (0.325–0.34); ML: male 0.135 (0.12–0.15), female 0.152 (0.15–0.155); MW: male 0.374 (0.322–0.426), female 0.51 (0.505–0.515); AL: male 0.658 (0.642–0.675), female 0.697 (0.685–0.71), GL: 1.03 (1.01–1.05), GW: 0.155 (0.15–0.16), PML: 0.055 (0.050–0.060).

##### Head 

(Figs 1–6). Anterior marginal carina very thick, with large and blunt marginal nodi; DHS 8–10 short fine to stout setae; DHS 15 long; occipital setae 21–22 thick setae of normal length; ventral subtemporal setae present; ocular and occipital nodi very well developed, connected with thick oculo–occipital and occipital carinae; maxillary palpi as in fig. 4; antennae (fig. 5) four segmented, pedicel large with short lateral process, bearing three stout sharp setae, flagellomere II long, oval with broad terminal disc; hypopharynx (fig. 6) very well developed.

**Figures 11–12.**
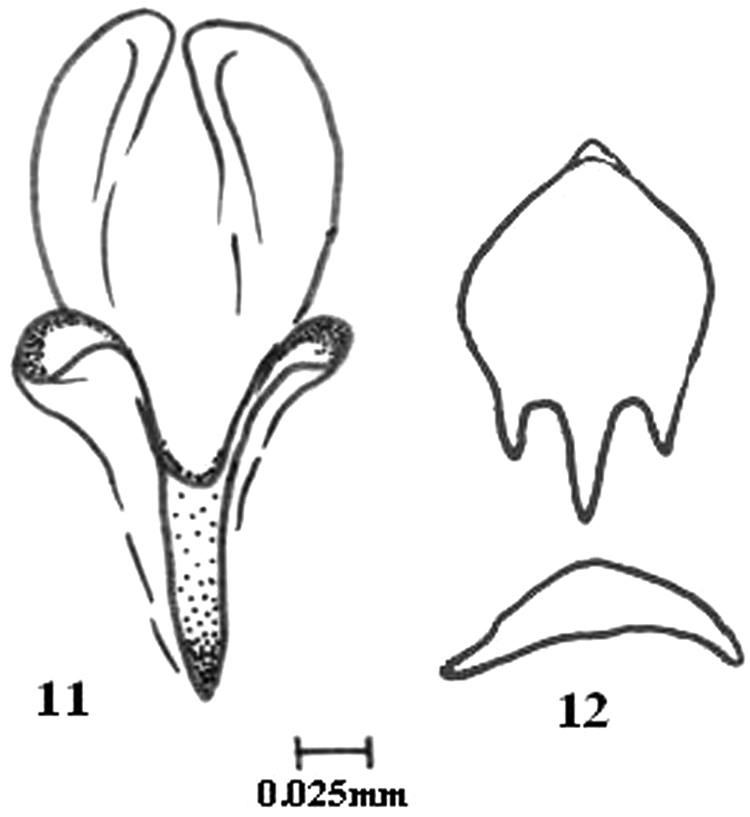
*Colpocephalum afrozeae* sp. n. **11** penis details **12** genital sclerite.

##### Thorax

(Figs 1–3, 7). Pronotal carina very thickly sclerotized; pronotal seta 2 minute peg-like setae; lateral to posterior margin of pronotum with four long and at least two short setae; prosternal plate (fig. 7) weakly developed, short, with posterior margin convex and lateral margins absent, one pair of small microsetae anterior to the plate present; posterior margin of metanotum straight, with 8–10 normal fine setae, arranged equally without any gape; femur III with two ctenidia.

##### Abdomen

(Figs 1–3, 8). Male. Tergal plates complete, marginal setae from tergite I–VIII: 12, 14, 17, 18, 16, 16, 15 and 13 respectively; anterior tergal setae scattered, ranging from 14–28 microsetae; postspiracular seta long on segment II, V–VII, shorter on segments III–IV and VIII; sternal setae in double rows on sternites I–VII: 15, 24, 16 (+ two large ctenidia on segment III; fig. 8), 24, 21, 20 and 16 respectively. Terminalia (Figs 1–2): Terminal segment comprises segments IX and X, posteriorly rounded; large tergal plate usually without anterior setae, latero–posterior margin with two long macrosetae and posterior margin bears four long macrosetae and two short fine setae; sternites VIII forming a short subgenital plate, bearing dense scattered small thin setae; anal margin almost straight.

Female. Tergites I and II complete, wide and long, tergites III–VIII divided, tripartite, narrow and short; tergocentral setae on segment I and II long; tergal marginal setae from I–VIII: 20, 20–22, 16–18, 18–20, 16–19, 17, 18 and 14–16 respectively; postspiracular setae long on II–III, VI–VIII; segment VIII with one pair of long, latero–anterior setae; sternite I developed, sternite II–VIII complete and well sclerotized; sternal setae small short to fine, scattered all over the plates; sternite III with two long ctenidia (fig. 8). Terminalia (fig. 9): Terminal segment widely rounded posteriorly; tergite IX divided, median piece triangular; posterior margin of lateral plates with small fine setae and two pairs of long macrosetae; anus narrow, transverse with tapering ends; anal fringes bear forty three stout microsetae in anterior fringe and forty seven to fifty fine curved setae in posterior fringe; vulval margin medially concave, with small thick, stout curved setae, gradually larger at latero–posterior ends.

##### Male genitalia

(Figs 10–12). Elongated; genital sclerite (fig. 12) short, with long and slightly curved latero-posterior points; genital lateral plates short and thick; basal plate thick and broad; median process long; penis (fig. 11) terminally narrow; parameres straight, tubular.

##### Remarks.

*Colpocephalum afrozeae* were collected from *Columba livia* on which *Colpocephalum turbinatum* has been reported previously. The two species of the genus *Colpocephalum* of *Columba livia* are different from each other. *Colpocephalum afrozeae* has the anterior margin of head broadly convex; anterior marginal carina thick; oculo-occipital carina thick; prothorax with two short marginal setae; femur III with two ctenidia; female tergite II with long tergocentral setae; postspiracular setae long on tergites II–III and VI–VIII; lateral plates of male genitalia very short; lateroposterior points of genital sclerite large and curved; median process reduced; female genital reticulation invisible; vulva medially concave; anus narrow and transverse.

*Colpocephalum afrozeae* has also some similarities with *Colpocephalum arfakiani* Price and Beer, but they have morphological differences, which consist of a thin anterior marginal carina; five long pronotal marginal setae; tergite II of female divided; tergite VIII with small triangular median piece; anal opening broad, with light fringe of short setae; male genital sclerite without latero-posterior points and long lateral plates are found in *Colpocephalum arfakiani* whereas the anterior margin very thick; four pronotal marginal setae long; tergite II of female complete; tergite VIII with large trapezoidal piece; anal opening narrow and transverse, with dense fringe of short setae in anterior margin and thick, long setae on posterior margin; male genital sclerite with long and curved latero-posterior points and short lateral plates are found in *Colpocephalum afrozeae*.

##### Etymology.

The present species is named after Mrs Hussan Afroze, mother of the first author.

#### Colpocephalum turbinatum

Denny

http://species-id.net/wiki/Colpocephalum_turbinatum

Fig. 13–16

Colpocephalum turbinatum Denny 1842: 198, 209, Harrison 1916: 56, Hopkins and Clay 1952: 84, Price andBeer 1963: 735, 736, 754, Hill and Tuff 1978: 308, 315, Lakshminarayana 1979: 80, Price et al. 2003: 102, 303, 304, 308.
Colpocephalum abruptofasciatum Mjöberg 1910: 36.
Colpocephalum ailurum Nitzsch (In Giebel) 1861: 522.
Colpocephalum bicinctum Nitzsch (In Giebel) 1861: 524.
Colpocephalum caudatum Giebel 1874: 261, Piaget 1880: 519, 1885: 125.
Colpocephalum dissimile Piaget 1880: 520, 1885: 119.
Colpocephalum intermedium Piaget 1880: 521.
Colpocephalum latifasciatum Piaget 1885: 130.
Colpocephalum osborni Carriker 1903: 172.
Colpocephalum oxyurum Nitzsch (In Giebel) 1861: 519.
Colpocephalum subflavescens Piaget 1880: 571.
Colpocephalum tricinctum Nitzsch (In Giebel) 1861: 524, Ansari 1951: 154.
Colpocephalum wernecki Orfila 1959: 477.
Neocolpocephalum gypae Qadri 1935: 229.
Neocolpocephalum tricinctum Eichler 1941: 374.
Vulturigogus eugenii Eichler and Zlotorzycka 1963: 207.
Vulturigogus femellus Eichler and Zlotorzycka 1963: 209.

##### Material examined.

91 males, 105 females, on *Columba livia* (Gmelin); Pakistan: Karachi; 21-V-2004, 23-IX-2007; leg. Naz.

**Figures 13–20.**
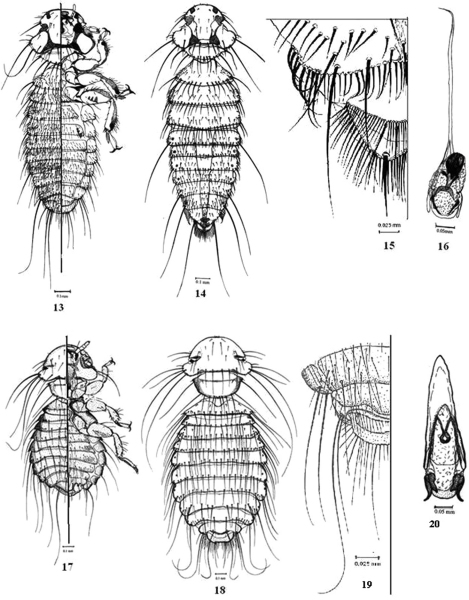
**13–16.** *Colpocephalum turbinatum* Denny **13** male dorso-ventral view **14** female dorsal view **15** female terminalia **16** male genitalia. **17–20.** *Hohorstiella lata* (Piaget) **17** male dorso-ventral view **18** female, dorsal view; **19**, female terminalia **20** male genitalia.

#### Hohorstiella lata

(Piaget)

http://species-id.net/wiki/Hohorstiella_lata

Fig. 17–20

Menopon latum Piaget 1880: 457.
Menopon giganteum Denny 1842: 225, Harrison 1916: 39.
Hohorstiella lata Eichler 1940: 362, Hopkins and Clay 1952: 173, Hill and Tuff 1978: 308, 310, Price et al. 2003: 111, 303.

##### Material examined.

25 males, 39 females, on *Columba livia* (Gmelin), *Streptopelia decaocta* (Frivaldszky); Pakistan: Karachi; 21-V-2004, 04-VIII-2006; leg. Naz. New record from Pakistan.

#### Hohorstiella streptopeliae

Eichler

http://species-id.net/wiki/Hohorstiella_streptopeliae

Fig. 21

Hohorstiella streptopeliae Eichler 1953: 169, Price et al. 2003: 111, 307.

##### Material examined.

4 females, on *Columba livia domestica* (Gmelin) (Fantail Pigeon breed); Pakistan: Karachi; 15-VII-2006; leg. Naz.

New record from Pakistan.

**Figure 21–25.**
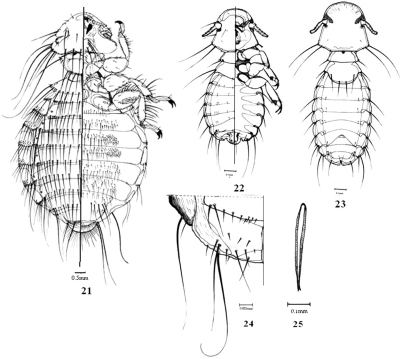
**21** *Hohorstiella streptopeliae* Eichler, female, dorso-ventral view. **22–25** *Campanulotes compar* (Burmeister) **22** male dorso-ventral view **23** female dorsal view **24** female terminalia **25** male genitalia.

### Suborder Ischnocera Kellogg, 1896

**Family Philopteridae Burmeister, 1838**

#### Campanulotes compar

(Burmeister)

http://species-id.net/wiki/Campanulotes_compar

Fig. 22–25

Goniocotes bidentatus Scopoli 1763: 385, Harrison 1916: 80.
Goniocotes compar Burmeister 1838: 431.
Goniocotes formosanus Sugimoto 1929: 25.
Goniodes compar Nitzsch 1818: 294, Denny 1842: 13, Giebel 1842: 12, Piaget 1842: 234, Neumann 1909: 31, Neveu–Lemaire 1919: 1116.
Campanulotes compar Keler 1939: 157, Hopkins and Clay 1952: 64, Ansari 1955: 48, Selimet al. 1968: 79, Hill and Tuff 1978: 309, 322, Tendeiro 1969: 380, 1978: 117, Lakshminarayana 1979: 70, Price et al. 2003: 160, 303.

##### Material examined.

51 males, 72 females, on *Columba livia* (Gmelin); Pakistan: Karachi, Hyderabad, Khairpur mir’s; 21-V-2004, 04-VIII-2006; leg. Naz.

New record from Pakistan.

#### Columbicola columbae

(L.)

http://species-id.net/wiki/Columbicola_columbae

Fig. 26–29

Pediculus columbae L. 1758: 614.
Lipeurus bacillus Nitzsch 1818: 215.
Lipeurus baculus Giebel 1866: 379, Kellogg 1896: 506, Neumann 1909: 30.
Lipeurus antennatus Giebel 1874: 213.
Philopterus baculus Nitzsch 1818: 293.
Phagopterus columbae Freire and Duarte 1944: 14.
Nirmus claviformis Olfers 1816: 90.
Nirmus filiformis Olfers 1816: 90.
Esthiopterum columbae Harrison 1916: 132.
Columbicola columbae Ewing 1929: 117, Ansari 1947: 259, Hopkins and Clay 1952: 86, Tendeiro 1960: 530, 533, Selim et al. 1968: 76, Hill and Tuff 1978: 309, 317, Lakshminarayana 1979: 82, Clayton and Price 1999: 675, Price et al. 2003: 166, 303.

##### Material examined.

48 males, 73 females, on *Columba livia intermedia* (Gmelin), *Columba livia neglecta* Hume; Pakistan: Karachi; 21-V-2004, 23-IX-2007; leg. Naz. New host record from Pakistan.

#### Columbicola tschulyschman

Eichler

http://species-id.net/wiki/Columbicola_tschulyschman

Fig. 30–33

Columbicola tschulyschman Eichler 1942: 28, Tendeiro 1960: 531, 571, Hopkins and Clay 1952: 88, Price et al. 2003: 168, 303.
Columbicola montschadskyi Blagoveshtchensky 1951: 308, Tendeiro 1965: 131.

##### Material examined.

5 males, 6 females, on *Columba livia neglecta* Hume; Pakistan: Karachi; 16-VIII-2007; leg. Naz.

New record from Pakistan.

**Figures 26–33.**
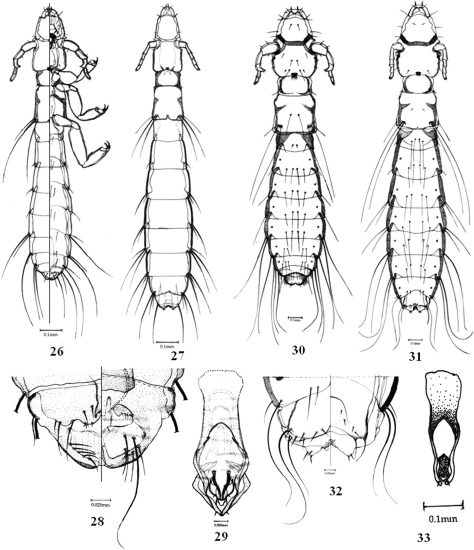
**26-29** *Columbicola columbae* (L.) **26** male dorso-ventral view **27** female dorsal view **28**, male terminalia dorso-ventral view **29** male genitalia. **30–33** *Columbicola tschulyschman* Eichler **30** male dorsal view **31** female dorsal view **32** female terminalia dorso-ventral view **33** male genitalia.

#### Turturicola salimalii

Clay & Meinertzhagen

http://species-id.net/wiki/Turturicola_salimalii

Turturicola salimalii Clay and Meinertzhagen 1937: 278, Ansari 1947: 260, Hopkins and Clay 1952: 360, Tendeiro 1965: 26, 48, Lakshminarayana 1979: 174, Price et al. 2003: 246, 307.

##### Material examined.

2 females, on *Columba livia* (Gmelin); Pakistan: Karachi; 16-VII-2005; leg. Naz.

New host record from Pakistan.

## Discussion

This study is the first survey of chewing lice of family Columbidae in Pakistan. Among the nine species found in this region, six species are recorded for the first time. Four of them, *Campanulotes compar*, *Colpocephalum turbinatum*, *Columbicola columbae* and *Hohorstiella lata*, are cosmopolitan (Emerson 1972, Ledger 1980, Mey 2003, Naz and Rizvi 2004, Naz et al. 2010).

Only two species of the genus *Colpocephalum* have been recorded from Columbidae, which are *Colpocephalum longicaudum* Nitzsch 1866 on *Streptopelia chinensis tigrina* (Temminck) and *Colpocephalum turbinatum* on *Columba livia* Gmelin (Price and Beer 1963, Price et al. 2003). Kellogg and Paine (1914) have reported *Colpocephalum longicaudum* from *Columba livia*. Price and Beer (1963) have recorded *Colpocephalum turbinatum* from various species of Falconiformes. Ansari (1951) reported *Colpocephalum turbinatum* from *Milvus migrans govinda* Sykes (Accipitridae: Falconiformes) with the synonym *Colpocephalum tricinctum*, in Lyallpur, Pakistan (Lakshminarayana 1979). Here, this species is reported from *Columba livia* in Karachi, Pakistan. Galloway and Palma (2008) showed that some species of lice can be overlooked for many decades even when they parasitize common hosts.

*Columbicola tschulyschman* is also a regular pigeon parasite. It is known from three species of *Columba* including *Columba livia neglecta*, which is also foundin Pakistan (Grimmett et al. 1999, Naz et al. 2010) and is probably still isolated from feral pigeons in Pakistan (Johnston 1996). There is no record of this louse species from feral pigeon (Adams et al. 2005).

The presence of *Hohorstiella streptopeliae* on *Columba livia* represents a case of straggling, because its type host is *Streptopelia turtur arenicola* (Hartlert) (Price et al. 2003). Ansari (1947) recorded *Turturicola salimalii* on three species of *Streptopelia* and on *Columba livia* from different regions of India, but he also collected this species from Passeriformes and Psittaciformes and suggested these hosts as likely stragglers.

## Supplementary Material

XML Treatment for Bonomiella columbae

XML Treatment for Colpocephalum afrozeae

XML Treatment for Colpocephalum turbinatum

XML Treatment for Hohorstiella lata

XML Treatment for Hohorstiella streptopeliae

XML Treatment for Campanulotes compar

XML Treatment for Columbicola columbae

XML Treatment for Columbicola tschulyschman

XML Treatment for Turturicola salimalii
